# Postoperative continuous non-invasive cardiac output monitoring on the ward: a feasibility study

**DOI:** 10.1007/s10877-020-00601-z

**Published:** 2020-10-22

**Authors:** C. E. King, A. Kermode, G. Saxena, P. Carvelli, M. Edwards, B. C. Creagh-Brown

**Affiliations:** 1 Medical Student, Faculty of Medicine, University of Southampton, Southampton, UK; 2 Surrey Perioperative Anaesthesia Critical Care Collaborative Research Group (SPACeR), Royal Surrey County Hospital, Egerton Road, Guildford, UK; 3 Department of Anaesthesia, Southampton General Hospital, Tremona Road, Southampton, UK; 4 Department of Clinical and Experimental Medicine, Faculty of Health and Medical Sciences, University of Surrey, Guildford, Surrey UK

**Keywords:** Postoperative hypotension, Haemodynamic, Feasibility, LIDCO CNAP, Fluid responsiveness

## Abstract

Postoperative hypotension is common (occurring in one third of patients) and is associated with worse clinical outcomes. The LiDCO CNAP (continuous non-invasive arterial pressure) device measures haemodynamics but has not been widely adopted in ward environments. Improved early detection of hypotension by CNAP might guide interventions to improve clinical outcomes. We aimed to find the proportion of patients who tolerated LiDCO CNAP for 12 h postoperatively, to unmask episodes of hypotension detected by continuous monitoring and to characterise the haemodynamic profile at the time of hypotension. In this feasibility study, patients undergoing major elective surgery were continuously postoperatively monitored using CNAP. Haemodynamic data gathered from CNAP, including nSVRI (nominal systemic vascular resistance index), nSVI (nominal stroke volume index), SVV (stroke volume variation) and blood pressure, were analysed using Microsoft Excel and GraphPad Prism 8. 104 patients (age (mean ± sd): 68 ± 14, male (56%)) had CNAP sited postoperatively. 39% tolerated the CNAP device for at least 12 h. Within the 104 patients a mean of 81.2 min of hypotension detected by CNAP was not detected by usual care. The proportion of low/normal/high nSVI was 71%, 27% and 2%, nSVRI was 43%, 17% and 40%, respectively. CNAP monitoring was not tolerated for 12 h in the majority of patients. There were many episodes of hypotension unmasked through continuous monitoring. Based on the advanced haemodynamic data provided it is possible that the underlying cause of a third of postoperative hypotensive episodes is vasodilation rather than hypovolaemia.

**Trial registry number**: NCT04010058 (ClinicalTrials.gov) Date of registration: 08/07/2019.

**Electronic supplementary material:**

The online version of this article (10.1007/s10877-020-00601-z) contains supplementary material, which is available to authorized users.

## Introduction

Patients having surgery under anaesthesia have comprehensive cardiorespiratory monitoring that continues into the early postoperative period [1]. Those patients who are staying in hospital after surgery, but are not requiring admission to critical care, have intermittent vital sign monitoring at intervals of one to six hours. Use of wearable technology with continuous measurements may help to detect early signs of clinical deterioration [2] as this often goes unnoticed for prolonged periods of time [3]. In an international study of ward patients, the commonest antecedent prior to major deterioration (cardiac arrest, unexpected intensive care unit (ICU) admission or death) was hypotension [3]. Postoperative hypotension was also associated with myocardial infarction (MI), acute kidney injury (AKI), increased mortality [4] and increased length of stay in hospital [5].

In a secondary analysis of the international VISION study of noncardiac surgical patients, postoperative hypotension was independently associated with increased risk of death or vascular events. Intraoperative hypotension was also common but was not significantly associated with mortality and vascular events after adjustment for postoperative hypotension [6]. Similarly, a secondary analysis of the POISE2 trial, showed that postoperative hypotension was significantly associated with a composite of myocardial infarction and death, even after adjustment for previous hypotension [7]. A single centre prospective cohort study with detailed characterisation of perioperative blood pressure showed that postoperative hypotension was strongly associated with myocardial injury, after adjusting for intraoperative hypotension [8].

Continuous measurement of blood pressure after abdominal surgery demonstrated that hypotension lasting at least 30 min was common (24%), and 21% of these cases were undetected by intermittent measurement of vital signs [4]. Postoperative hypotension is commonly assumed to be caused by hypovolaemia and is often treated with rapid administration of an intravenous fluid bolus (IVFB). However, a normal-to-high nSVI accompanied by a low nSVRI may indicate vasodilatory hypotension, and in a mixed population of medico-surgical ward patients this haemodynamic profile has been shown to be common during episodes of hypotension [9, 10]. Given that IVFB may be ineffective, or even harmful [4], more targeted treatment of postoperative hypotension might lead to improved clinical outcomes.

There are a range of non-invasive cardiac monitoring devices that can be used on the ward, each relying on different technologies including thoracic bioreactance, plethysmography, and impedance cardiography [11, 12]. The LiDCO CNAP device is a non-invasive continuous cardiac output monitor that can provide additional haemodynamic data. It uses the vascular unloading technique described by Peňáz, with an inflatable dual finger cuff and plethysmograph. Finger blood volume (measured by light absorption) is kept constant in the face of varying arterial pressure by a fast-acting servo system which inflates and deflates the cuff in time with arterial pulsations. The pressure waveform in the cuff correlates to the pressure waveform of the finger arteries, and through calibration with a conventional arm blood pressure cuff gives a constant arterial pressure readout. This is analysed by a proprietary algorithm to give an estimate of cardiac stroke volume and other variables.[13]. It has been evaluated in a range of surgical populations [14–17], a post-operative cohort [18], and a haemodynamically unstable critical care population [19]. To our knowledge no non-invasive cardiac output monitors have been studied in exclusively postoperative non-cardiac surgical patients.

We conducted this prospective observational study to gain information to assist in designing a trial that will test the hypothesis that additional information about postoperative haemodynamics can improve clinical outcomes. Our primary aim was to establish if it is feasible for awake postoperative patients to wear the CNAP for up to twelve hours. Secondary aims included a determination of how commonly episodes of hypotension were detected with CNAP but missed with conventional intermittent blood pressure measurements, and by characterising the haemodynamic profile of episodes of hypotension whether they are characteristic of hypovolaemia or not.

## Methods

### Study design

This was a prospective observational cohort study conducted at Royal Surrey County Hospital, a medium-sized community hospital in the UK. We enrolled patients aged 18 and over scheduled to undergo non-cardiac surgery requiring an overnight stay in hospital. Patients were excluded if admission to the intensive care unit was planned, if the patient had impaired circulation of the hands, or if they declined to participate. Written informed consent was obtained from each subject on the morning before their operation.

Participants had the CNAP sited after emergence from anaesthesia in the post-anaesthetic care unit (PACU) and were asked to continue wearing it for up to 12 h or until they asked for its removal. Once patients had recovered from anaesthesia and a bed had become available, both the patient and CNAP were moved to the ward (unless the device had been removed before this point). The CNAP device collected continuous heart rate and blood pressure data, from which it estimated a nominal cardiac output and derived variables including nominal stroke volume index (nSVI) and nominal systemic vascular resistance index (nSVRI). Only the patients’ blood pressure and heart rate was displayed on the monitor and this was covered by a paper sheet, only removed when usual observations would take place. Hiding the monitor in this way ensured that the continuous haemodynamic data from the CNAP would not alter clinical management.

### Data collection

Patient demographics were collected using medical notes and if intravenous fluids (IVF) were given by the clinical team the details were recorded from the drug chart. Surgery and anaesthetic data were obtained from anaesthetic charts or print outs. Hypotension was defined with reference to the recent Peri-Operative Quality Improvement group (POQI) statement as a systolic blood pressure of less than 90 mmHg [5]. Standard clinical monitoring and observations were conducted by attending clinical staff in line with routine care and recorded using paper (PACU) and the VitalPAC system (wards), providing data for subsequent analysis. Duration of hypotension for intermittent readings was defined as the length of time passed, from the minute a systolic blood pressure below 90 mmHg was recorded, until a reading above 90 mmHg had been recorded.

Length of stay was calculated by assuming one overnight stay postoperatively equated to 1 day. Length of stay (LOS) was determined either by day of discharge or the final day of data collection (19th November 2019) whichever came sooner. Clinical outcomes (acute kidney injury (AKI), myocardial infarction (MI) and hospital length of stay (LOS)) were collected weekly by reviewing ICE pathology reports and APAS (Allscripts patient administration system). AKI was defined using the KDIGO classification, increase in creatine was calculated using a pre-operative result as baseline, up to 1 year preoperatively, and the highest postoperative result, up to 7 days postoperatively. An increase in creatinine > 1.5 times baseline within 7 days was classed as an AKI. MI was defined as Troponin T above the normal range (> 14 ng/L). Clinical outcomes were only assessed during a patient stay in hospital or up to the end date of the study, whichever came sooner. Individual patient predicted mortality risk within 30 days of surgery was estimated using the Surgical Outcome Risk Tool (SORT) calculator at www.sortsurgery.com.

### Data analysis

Haemodynamic data from the CNAP were exported and viewed using LIDCOview PRO software, from which exported data were entered into Microsoft Access. If there was a > 20% difference in systolic blood pressure between continuous reading and blood pressure cuff during recalibration, data from the prior 10 min were excluded. This was to exclude artefactual data due to patient movement. Data were analysed using GraphPad Prism version 8.

### Statistical analysis plan

Categorical data are presented as the number of patients and a percentage. Data were visually inspected and assessed using GraphPad Prism version 8 to determine normality. Parametric data are displayed as a mean and standard deviation, non-parametric data are displayed as a median and interquartile range. Statistical differences in hypotension detected in CNAP compared to usual care was assessed in GraphPad Prism version 8 using Wilcoxon signed rank test. Variables were considered statistically significant if p < 0.05.

## Results

### Patients

One hundred and sixteen patients were recruited between 2nd September and 8th November 2019. Most had at least one co-morbidity, with hypertension being the most common; 29% of patients had received anti-hypertensive medication on the day of surgery, the most common being beta blocker. Nearly half of all patients experienced at least one episode of intra-operative hypotension, and there was widespread use of intra-operative vasopressors. See: Table 1.

**Table 1:** Patient demographics, all averages are expressed as a mean except age which is non-parametric data

Male n (%)	58 (56)
Age yrs (median (IQR))	69.5 (14.7)
Mean blood pressure taken > 12hrs preoperatively, mean (min–max)	135/78 (102/58 -175/101)
Height (cm), mean (min–max)	170 (146–189)
Weight (kg), mean (min–max)	82 (48–139)
Co-Morbidities n(%)	
Hypertension	47 (45)
Cancer	28 (27)
Ischaemic heart disease	14 (13)
Rheumatological disease	9 (9)
Atrial fibrillation	9 (9)
Chronic obstructive pulmonary disease	5 (5)
Chronic kidney disease	2 (2)
Anti-hypertensive medication (taken on day of surgery) n(%)	
ACEI	10 (10)
ARB	1 (1)
CCB	5 (5)
Beta blocker	12 (12)
Diuretic	7 (7)
Alpha blocker	1 (1)
None	74 (71)

*ACEI* Angiotensin converting enzyme inhibitor, *ARB* Angiotensin receptor blocker, *CCB* Calcium channel blocker

Most patients underwent orthopaedic surgery with volatile anaesthesia and a spinal neuraxial blockade. See: Table 2. More information with regards to surgery performed can be found attached as an electronic supplementary material (Table S1 in ESM_1.docx).

**Table 2:** Details of anaesthesia and surgery

Estimated mortality within 30 days of surgery	0.76%
Surgery type n(%)	
Orthopaedic	56 (53)
Gynae-oncology	3 (3)
Urology	32 (31)
General	13 (13)
Length of anaesthesia(mins), mean(min–max)	164 (45–585)
Anaesthesia type n(%)	
Total intravenous anaesthesia	28 (27)
Volatile	61 (59)
Both	2 (2)
Neither	13 (13)
Neuraxial blockade n(%)	
Spinal with general anaesthetic	60 (58)
Spinal without general anaesthetic	13 (13)
Epidural	0 (0)
None	31 (30)
Intra-operative vasopressors n(%)	
Bolus (epinephrine)	14 (13)
Infusion (phenylephrine)	40 (38)
Both	12 (12)
Neither	38 (37)
Episodes of intra-operative hypotension (Systolic Blood Pressure < 90 mmHg) n(%)	
None	61 (59)
1	9 (9)
> 1	34 (33)

Averages expressed as a mean

*TIVA* Total intravenous anaesthesia

### Outcomes

Out of the 116 patients recruited for the study, 104 of these patients had the device sited in recovery.

#### Primary outcome

**Fig. 1:**
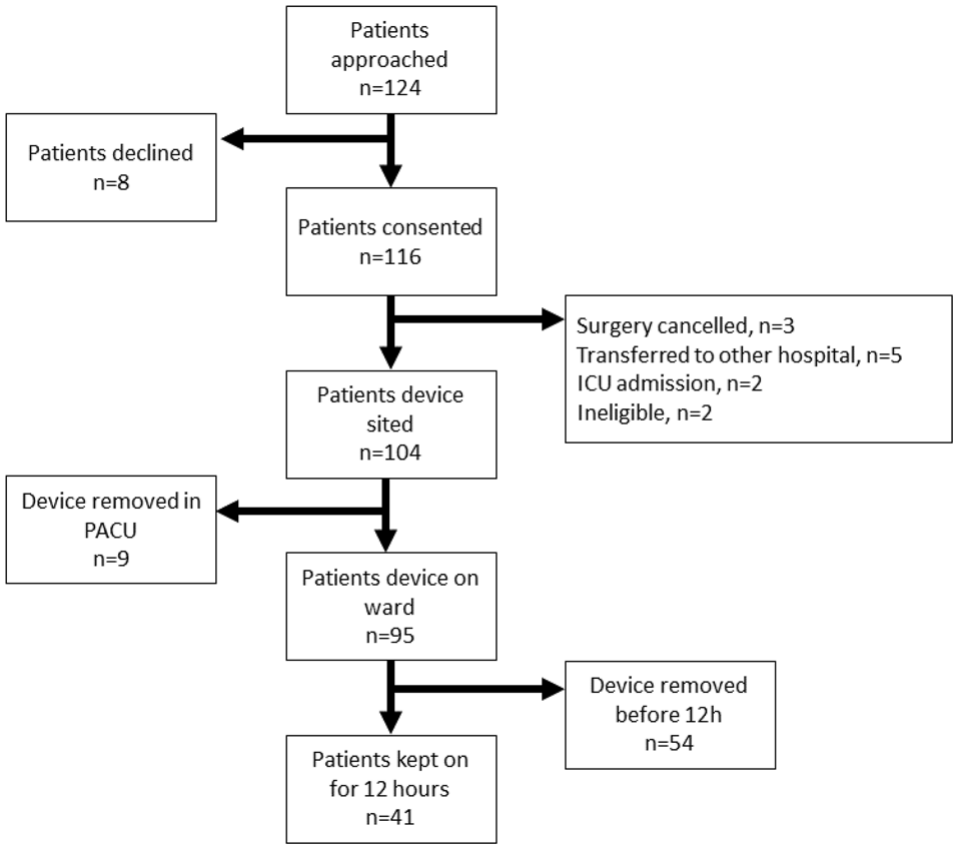
CONSORT diagram

41/104 (39%) of participants who had the device sited kept it on throughout the observation period (at least 12 h) and provided usable data (Fig. 1). If CNAP was tolerated but haemodynamic variables were not recorded (e.g. due to machine error) this would have been classed as unusable data, but this did not occur.

**Fig. 2:**
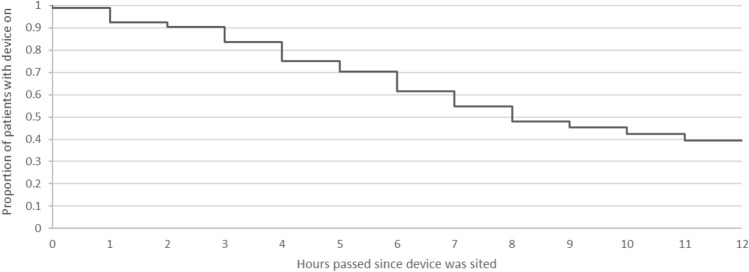
CNAP survival analysis. Demonstration of the gradual decline in use of the CNAP device over time

The proportion of patients wearing the device gradually declined over time (Fig. 2). For logistic reasons we did not have investigators present at the bedside throughout the 12 post-operative hours and therefore the reasons for device removal or failure to obtain data from the device were not always readily apparent. Research staff observation and patient feedback has led us to believe the following factors were important. Firstly, patient discomfort from the device irritating the delicate hands of the patients—often the plastic frame on the finger cuff would cause redness in the third interdigital fold. Secondly arm cuff inflation (when the device automatically recalibrates) could have been inconvenient for participants trying to sleep. Thirdly, despite education of the ward staff there was limited expertise in troubleshooting during the night and device malfunction (for example loose connection between the monitor and finger cuff would cause measurements to stop and mimic patient removal of the device) wasn’t necessarily optimally resolved and tended to lead to the device being removed.

#### Secondary outcomes

The incidence of hypotension and the duration of hypotension were both greater when measured with the CNAP than with standard measurements (see Table 3). When including all study patients, the overall duration of hypotension was longer when measured with the CNAP compared to standard measures, on the PACU and the ward. Considering only those patients who had at least one episode of hypotension (as defined by CNAP readings), duration of hypotension was also greater when measured with the CNAP than standard measures, on the PACU and the ward.

**Table 3:** Duration of hypotension detected by CNAP compared to standard intermittent care using data from all 104 participants

	CNAP-detected hypotension	Standard care-detected hypotension	p-value
Number of patients with ≥ 1 episode of hypotension:			
Occurring within PACU, n (% of all participants)	27 (26)	2 (1.9)	< 0.0001
Occurring within ward, n (% of all participants)	46 (48)	3 (3.2)	< 0.0001
Occurring at any point, n (% of all participants)	56 (54)	5 (4.8)	< 0.0001
Total duration of hypotension (per patient using all 104 study participants):			
Occurring within PACU, mins (% of total monitoring period)	7.2 (4.4)	0.2 (0.1)	< 0.0001
Occurring within ward, mins (% of total monitoring period)	40.1 (8.9)	0.7 (0.2)	< 0.0001
Occurring at any point, mins (% of total monitoring period)	43.7 (7.6)	0.9 (0.2)	< 0.0001
Total duration of hypotension (per patient only using participants with ≥ 1 episode of CNAP-detected hypotension):			
Occurring within PACU, mins (sd)	27.4 (24.7)	0.7 (3.0)	< 0.0001
Occurring within ward, mins (sd)	82.7 (97.2)	1.5 (8.9)	< 0.0001
Occurring at any point, mins (sd)	81.2 (94.0)	1.6 (8.2)	< 0.0001

**Fig. 3:**
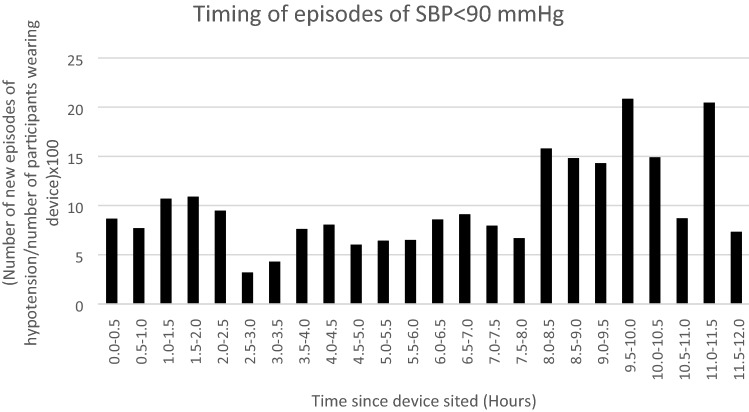
This graph shows when new episodes of hypotension (SBP < 90 mmHg) occurred during the first 12 h of observation in patients still wearing the CNAP device

The mean lowest systolic blood pressure during an episode of hypotension was 71 mmHg (34-90 mmHg). The mean time of day in which the device was sited was 12:55 (range 10:10–18:05hrs). The most common time for episodes of hypotension was between 8 and 11.5 h after the monitoring was commenced (Fig. 3). New episodes of hypotension occurring in each half hour period ranged from 3–21, proportionate to the number of participants still wearing the device.

When comparing baseline characteristics and clinical outcomes between participants who experienced at least one episode of hypotension and those who did not there was only one notable finding (Table S2 in ESM_1.docx). There was a statistically significant difference in baseline blood pressure with the group experiencing at least one episode of hypotension having a lower mean systolic and diastolic blood pressure. There was no difference in the proportion with a history of hypertension between the groups or in those who had taken their antihypertensive medication on the day of surgery in the group who had at least one episode of hypotension. There was no difference in clinical outcomes between the groups. The study was not powered to demonstrate differences in clinical outcomes.

**Fig. 4:**
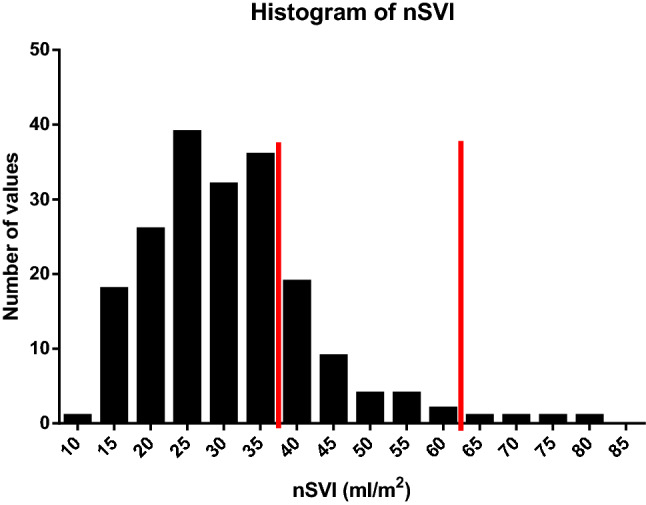
The distribution of nSVI recorded during any episode of CNAP-defined hypotension (SBP < 90 mmHg) with the red lines separating low (< 36 ml/m2) from normal from high sSVI (> 64 ml/m2)

Considering all periods of hypotension, the distribution of nSVI showed a skewed distribution mostly at the low end of the spectrum (median (IQR), 29 ml/m2 (23–36 ml/m2). All defined normal ranges are arbitrary but using manufacturer defined ranges, low was < 36 ml/m^2^ and high was > 64 ml/m^2^. The distribution of nSVI during hypotension was low (71%), normal (27%) and high (2%), see: Fig. 4.

**Fig. 5:**
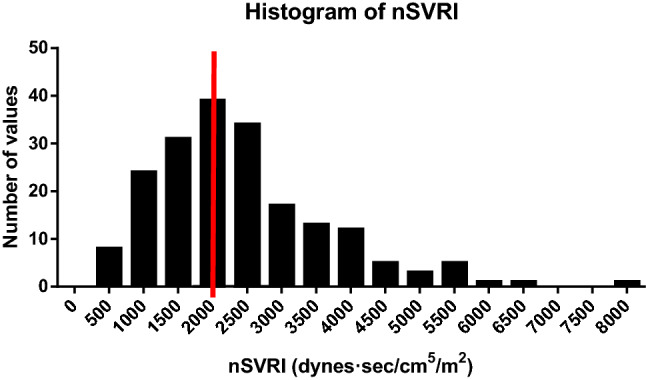
The distribution of nSVRI recorded during any episode of hypotension (SBP < 90 mmHg) with the red line separating low (< 2000 dynes.sec/cm^5^/m^2^) from normal/high

The CNAP device can also estimate the systemic vascular resistance index (SVRI) when paired with mean arterial pressure and notional RAP (right atrial pressure). During episodes of hypotension the median SVRI was (IQR), 2142 dynes.sec/cm^5^/m^2^ (1470–2904 dynes.sec/cm^5^/m^2^). Episodes of hypotension were associated with a low SVRI (< 2000 dynes.sec/cm^5^/m^2^) on 44% of occasions, consistent with vasodilatation (see: Fig. 5).

The average length of stay in hospital was 3.1 days. No myocardial infarctions occurred postoperatively, however one recruit (0.1%) experienced stage 1 AKI. There was no use of vasopressor drugs following surgery.

## Discussion

This study demonstrates that postoperative use of the CNAP device is well tolerated in the early postoperative period (on PACU) but only 39% of participants can tolerate continuous observations for 12 h following surgery. Refinements in the technology to make the device less bulky and more comfortable, and adjusted patient and staff expectations and familiarity could improve duration of use [12]. This contrasts with other continuous monitors like wireless chest patches that have been found to be well tolerated and comfortable [3]. A problem common to all monitoring devices is artifacts and false alarms, and the consequent alarm fatigue – which developments in machine learning may help overcome [12].

Hypotension was far more commonly detected with continuous measurements as compared to intermittent: using the CNAP in the ward environment detected systolic blood pressure of < 90 mmHg for 8.9% of the total observation time, compared with 0.1% with standard measures. Similarly, 53.8% of all participants had at least one episode of hypotension with the CNAP, compared to 4.8% with standard measurements. This agrees with other studies that showed non-invasive cardiac output monitors detect more hypotension than standard measures [4]. Detecting episodes of postoperative hypotension that may go undetected by intermittent measures may lead to quicker treatment, less associated organ injury, and therefore decreased length of stay [4].

The peak incidence of episodes of hypotension was between 8 and 11.5 h after placing the device. Given the time that surgery finishes, this means that the peak onset of hypotension is the early evening and into the night. This finding needs to be validated in other studies as it differs from other descriptions where the peak incidence was earlier [20]. Staffing levels tend to be at their lowest over night and this could lead to delays in assessment and management. Collection of data about sleep would have allowed us to determine if the onset of sleep coincided with the onset of hypotension, although this is a well-recognised relationship.

Postoperative hypotension can have several causes, hypovolaemia (this can be due to blood loss or inadequate fluid replacement) or vasodilation (this can be exacerbated by sympathetic blockade using epidurals). A significant proportion (29%) of episodes of hypotension were associated with normal or high nominal stroke volume index suggesting that hypovolaemia may not have been the primary cause of hypotension. Accordingly, a low systemic vascular resistance index was present in 44% of all episodes of hypotension. Together these results imply that during routine low-risk elective surgery, vasodilatation may commonly contribute towards hypotension. Previous research has found non-invasive cardiac output monitors may help to guide therapeutic intervention [11]. Systemic vasodilatation (as a consequence of the inflammatory response to surgery, sympathetic blockade or postoperative rewarming of the patient) would be expected to lower afterload, and also to reduce preload through the redistribution of circulating volume from the ‘stressed’ to ‘unstressed’ compartments. This would manifest as a low nSVI and reduced SVRI.

The limitations to this study include the potential inaccuracy of the CNAP in measuring the continuous arterial blood pressure given that the participants may have been moving during acquisition. This could account for discrepancies between blood pressure recorded via standard measures and those recorded by CNAP. We did not predict these discrepancies and therefore have not obtained quantitative data regarding the frequency or magnitude at which these discrepancies occurred. Furthermore, some inaccuracy may stem from the limitations of the non-calibrated LiDCO algorithm in deriving the cardiac output and therefore stroke volume (nSVI), and the necessary assumptions in calculation of the vascular resistance (nSVRI). However, studies have shown no statistical difference between CNAP and standard measures [14, 16] and concluded CNAP to be a reliable continuous blood pressure monitor [18]. On the contrary, one study suggesting the agreement between CNAP and standard measures decreases during hypotensive episodes.[15]. Therefore, discrepancies between CNAP recordings and intermittent recordings may need to be evaluated in further studies.

Given the limited duration of usage we did not acquire the desired duration of measurement in the majority of participants. We chose a convenience sample of patients and deliberately avoided those destined for ICU where invasive cardiac output monitoring is standard care. An unintended consequence of this was that a limited number of IVF boluses were administered, and therefore we were unable to draw conclusions on the change in measured values in response to fluid resuscitation. The lack of IVF boluses was likely due to the low prevalence of hypotension detected by standard measures. An additional limitation of not having a member of the team with the patient at all times was that some of the measurements might have been taken during patient movement or other physical reason for inaccurate measurements and this would not have been detected. Device troubleshooting and encouragement of continuous use could also not be carried out, however this mirrors real clinical practice. There was a variable delay of up to one hour from when the patient arrived in PACU to when the CNAP device was sited. This may have reduced detection of episodes of hypotension. We were not able to obtain data on why the device was removed before 12 h had passed and this is a limitation.

As a feasibility and acceptability study our sample size is adequate, and our inclusion criteria covered a wide range of common surgical conditions. Other strengths include having up to six CNAP devices available which facilitated a rapid recruitment rate.

The implications of this study are threefold. Firstly, these data are consistent with previous evidence suggesting that continuous BP measurement signals hypotension that is not detected by conventional intermittent measures [4]. The clinical utility of increased detection of postoperative hypotension remains to be fully determined but given the prognostic significance of prolonged postoperative hypotension, earlier detection and treatment has the potential to improve clinical outcomes [2]. Secondly, CNAP technology can be worn in the early postoperative period but is less well tolerated after this period. Finally, and potentially most importantly, systemic vasodilatation may be a very common contributing factor to postoperative hypotension and the implication of this is that treatment with an IVF bolus may be less effective than an appropriate dose of vasoconstrictor drug. This alternative approach warrants prospective evaluation in an appropriate designed clinical trial.

## Conclusions

Less than half of the patients kept the CNAP device in place until the conclusion of the 12 h period of continuous postoperative monitoring; leading us to conclude that the technology needs refining for this indication. However, many episodes of early postoperative hypotension were unmasked and a large portion of episodes of hypotension were associated with haemodynamic profiles that are not characteristic of hypovolaemia, and more in keeping with vasodilatory hypotension. Therefore the prevailing treatment paradigm of intravenous fluid boluses could potentially be improved upon. Continuous non-invasive haemodynamic monitoring in the early postoperative period could improve patient outcome.

## Electronic supplementary material

Below is the link to the electronic supplementary material.

Supplementary file1 (DOCX 18 kb)

## Data Availability

Data are available from the authors upon reasonable request.

## References

[1] Checketts MR (2016). Recommendations for standards of monitoring during anaesthesia and recovery 2015: Association of Anaesthetists of Great Britain and Ireland. Anaesthesia.

[2] Downey C, Randell R, Brown J, Jayne DG (2018). Continuous Versus Intermittent Vital Signs Monitoring Using a Wearable, Wireless Patch in Patients Admitted to Surgical Wards: Pilot Cluster Randomized Controlled Trial. J Med Internet Res.

[3] Kause J (2004). A comparison of antecedents to cardiac arrests, deaths and emergency intensive care admissions in Australia and New Zealand, and the United Kingdom—the ACADEMIA study. Resuscitation.

[4] Turan A (2019). Incidence, severity, and detection of blood pressure perturbations after abdominal surgery: a prospective blinded observational study. Anesthesiology.

[5] Farley KX (2019). The influence of modifiable, postoperative patient variables on the length of stay after total hip arthroplasty. J Arthroplasty.

[6] Roshanov PS (2017). Withholding versus continuing angiotensin-converting enzyme inhibitors or angiotensin II receptor blockers before noncardiac surgery. Anesthesiology.

[7] Sessler DI (2018). Period-dependent associations between hypotension during and for four days after noncardiac surgery and a composite of myocardial infarction and death: a substudy of the POISE-2 Trial. Anesthesiology.

[8] Liem, V. F. B. et al. Postoperative Hypotension after Noncardiac Surgery and the Association with Myocardial Injury. *Anesthesiology.*(2020) 10.1097/ALN.0000000000003368 32487822

[9] Eyeington CT (2019). Rapid response team review of hemodynamically unstable ward patients: The accuracy of cardiac index assessment. J Crit Care.

[10] Eyeington CT (2019). Non-invasive continuous haemodynamic monitoring and response to intervention in haemodynamically unstable patients during rapid response team review. J resuscitation.

[11] Saugel B, Cecconi M, Wagner JY, Reuter DA (2015). Noninvasive continuous cardiac output monitoring in perioperative and intensive care medicine. Br J Anaesth.

[12] Michard F (2020). A glimpse into the future of postoperative arterial blood pressure monitoring. Br J Anaesth.

[13] Fortin J (2006). Continuous non-invasive blood pressure monitoring using concentrically interlocking control loops. Comput Biol Med.

[14] Jeleazcov C (2010). Precision and accuracy of a new device (CNAPTM) for continuous non-invasive arterial pressure monitoring: assessment during general anaesthesia. Br J Anaesth.

[15] Ilies C (2012). Investigation of the agreement of a continuous non-invasive arterial pressure device in comparison with invasive radial artery measurement. Br J Anaesth.

[16] Hahn R, Rinösl H, Neuner M, Kettner SC (2012). Clinical validation of a continuous non-invasive haemodynamic monitor (CNAPTM 500) during general anaesthesia. Br J Anaesth.

[17] Biais M (2010). Continuous non-invasive arterial pressure measurement: Evaluation of CNAPTM device during vascular surgery. Ann Fr Anesth Réanimation.

[18] Jagadeesh AM, Singh NG, Mahankali S (2012). A comparison of a continuous noninvasive arterial pressure (CNAPTM) monitor with an invasive arterial blood pressure monitor in the cardiac surgical ICU. Ann Card Anaesth.

[19] Monnet X (2012). Prediction of fluid responsiveness by a continuous non-invasive assessment of arterial pressure in critically ill patients: comparison with four other dynamic indices. Br J Anaesth.

[20] Cirbian J (2014). Incidence and timing of hypotension after transcervical carotid artery stenting: Correlation with postoperative complications. Catheter Cardiovasc Interv.

