# Supplementary material for: Postoperative continuous non-invasive cardiac output monitoring on the ward: a feasibility study
Source: J Clin Monit Comput. 2020 Oct 22;35(6):1349–56. doi: 10.1007/s10877-020-00601-z (PMC8542541; doi:10.1007/s10877-020-00601-z)
Supplement: Supplementary file 1 — Supplementary file1 (DOCX 18 kb) [file 10877_2020_601_MOESM1_ESM.docx]

Table S1

| **Surgical procedure performed** | **Frequency** |
| --- | --- |
| **Orthopaedic** |  |
| Anterior repair spinosacral fixation | 1 |
| Total hip replacement | 22 |
| Correction of clubbed foot | 1 |
| Discectomy | 2 |
| Total ankle replacement | 2 |
| Total knee replacement | 17 |
| Tibialis anterior tendon transfer peroneus longus tendinosis spacer change | 1 |
| Unicompartmental knee replacement | 1 |
| Spinal decompression | 2 |
| Open reduction internal fixation of ankle | 3 |
| Repair fractured neck of femur | 2 |
| Osteotomy and subtalar fusion | 1 |
| Revision of total knee replacement | 1 |
| **General** |  |
| ileostomy reversal | 3 |
| Laparoscopic left colon resection | 1 |
| Sigmoid colectomy | 2 |
| Staging laparoscopy | 1 |
| Hartman’s reversal | 2 |
| Gastroenterostomy | 1 |
| Hemicolectomy | 2 |
| Reduction mammoplasty | 1 |
| **Gynae-oncology** |  |
| Bilateral Salpingoophorectomy and laparoscopic hysterectomy | 3 |
| **Urology** |  |
| Transurethral resection of bladder tumour | 3 |
| Transurethral resection of bladder tumour and cystoscopy | 2 |
| Transurethral resection of prostate | 8 |
| Transurethral resection of prostate and cystoscopy | 2 |
| Robotic prostatectomy | 17 |

Table S2

The baseline characteristics and outcome (mean length of stay) for the participants divided into those who experienced at least one episode of hypotension and those who did not. Statistical differences and normality were assessed in GraphPad Prism version 8 using unpaired T test.

|  | Participants experiencing at least one episode of hypotension (SBP<90mmHg)  N=55 | Participants not experiencing an episode of hypotension (SBP<90mmHg)  N=49 | p |
| --- | --- | --- | --- |
| Age, years (min-max) | 68 (31-95) | 69 (38-92) | 0.37 |
| Male n(%) | 28(50.9) | 30(61.2) | 0.43 |
| Height, cm (min-max) | 170 (146-186) | 170 (155-189) | 0.96 |
| Weight, kg (min-max) | 80 (48-118) | 85 (52-139) | 0.17 |
| Blood pressure (min-max) | 131/76 (104/61-175/95) | 141/81 (102/58- 169/101) | <0.01/  <0.01 |
| co-morbidities n(%) | 32(58.2) | 24(49.0) | 0.50 |
| Specific co-morbidity:  hypertension n(%) | 28(50.0) | 25(51.0) | 0.94 |
| Antihypertensive medication taken on day of surgery n(%) | 20(36.4) | 10(20.4) | 0.37 |
| Length of anaesthesia, mins (min-max) | 151.5 (45-585) | 144.7 (45-390) | 0.69 |
| Spinal neuraxial blockade n(%) | 40 (72.7) | 33 (67.3) | 0.62 |
| Length of stay, days (min-max) | 3.0 (1-18) | 3.2 (1-15) | 0.75 |
